# Demographic Disparities in Stroke Occurrence: Insights From an Integrative Review of Emerging Trends

**DOI:** 10.1002/brb3.70575

**Published:** 2025-08-04

**Authors:** Gudisa Bereda

**Affiliations:** ^1^ Pharmacy, All Africa Leprosy, Tuberculosis and Rehabilitation Training Centre Addis Ababa Ethiopia

**Keywords:** age, demographic disparities, ethnicity, geographic trends, prevalence, socioeconomic status, stroke

## Abstract

**Background and Objectives:**

Stroke continues to be a major global health challenge, with notable differences in its occurrence across various demographic groups. This review aimed to investigate trends in stroke occurrence, focusing on disparities related to age, sex, ethnicity, socioeconomic status, and geographic location.

**Methods:**

A thorough review of the literature was conducted using prominent databases such as PubMed, Web of Science, Google Scholar, and Scopus. Studies published between 2015 and 2025 that examined stroke in relation to demographic factors were included. Articles were selected based on relevance, study design, and the rigor of the research methodology. Both qualitative and quantitative studies were included in the synthesis. The data were synthesized qualitatively to highlight significant trends.

**Results:**

This review revealed that age is a key factor in the occurrence of stroke. Stroke prevalence increases with age, particularly in individuals aged 65 years and older. Gender differences in stroke risk are evident—men tend to have higher stroke risks at younger ages, whereas women face a greater risk later in life, especially after menopause. Ethnic disparities are also significant, with African Americans and Hispanics experiencing a higher risk of stroke compared to Caucasians. This increased risk is influenced by genetics, socioeconomic factors, and a higher prevalence of hypertension and diabetes.

**Conclusion:**

Demographic disparities significantly affect both the occurrence and outcome of stroke. Addressing these disparities requires comprehensive approaches, including enhanced access to preventive care, culturally sensitive health education, and targeted policies aimed at high‐risk groups.

## Graphical abstract

Stroke prevalence increases dramatically among individuals aged 65 years and older, highlighting the significant impact of aging on stroke risk. Gender differences are also apparent, with men experiencing more strokes at younger ages, while women face an increased risk after menopause. Ethnic disparities show that African Americans and Hispanics are at higher risk of stroke, primarily due to a greater prevalence of hypertension and diabetes within these populations. In addition, socioeconomic and geographic factors contribute to worse stroke outcomes, particularly in areas with limited access to preventive care services. Tackling these disparities requires equitable policies and targeted educational efforts for vulnerable groups.

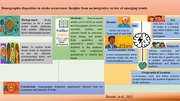

## 1 Introduction

A preprint version of this manuscript has been previously published by Bereda (2025) on the MDPI preprint server with Doi: https://doi.org/10.20944/preprints202504.0028.v1. Stroke remains one of the leading causes of morbidity and mortality worldwide, exerting a significant burden on individuals, families, and healthcare systems (Bereda 2025). Stroke, characterized by the sudden loss of neurological function due to either ischemic or hemorrhagic events, is becoming increasingly prevalent, particularly in low‐ and middle‐income countries where healthcare systems are often under‐resourced (Saini et al. 2021). In recent decades, significant advances have been made in understanding the epidemiology of stroke, with emerging data highlighting shifting trends in its occurrence, prevalence, and associated risk factors. Nevertheless, the burden of stroke remains unevenly distributed across demographic groups, revealing persistent disparities (Prust et al. 2024; de Havenon et al. 2023). These differences are shaped by factors such as age, sex, ethnicity, socioeconomic status (SES), geographic location, and access to healthcare. This review examines the existing literature to highlight the key trends in stroke epidemiology and demographic disparities. It also explores the influence of lifestyle changes, urbanization, aging populations, and advancements in diagnostic tools on stroke prevalence.

## 2 This Study Adds

This review provides a comprehensive overview of recent trends and data concerning demographic disparities in stroke occurrence, offering a comprehensive perspective. It emphasizes lesser‐explored factors, such as the role of intersectionality, including the combined effects of race, gender, and SES. In addition, it presents new insights into emerging trends, such as the rising prevalence of stroke among younger populations and in low‐income and middle‐income countries.

## 3 Methods and Materials

### 3.1 Study Design

This integrative review employed a structured approach to examine recent trends in stroke occurrence, with a focus on demographic disparities by merging both qualitative and quantitative studies.

### 3.2 Research Questions

The review was guided by three central research questions:

1. How do age, sex, and race/ethnicity influence the prevalence of ischemic and hemorrhagic strokes?
2. What is the relationship between socioeconomic status and the prevalence of stroke across different demographic groups?
3. What is the impact of geographic location, particularly urban versus rural settings on stroke prevalence and outcomes, with a focus on healthcare access and behavioral factors?

### 3.3 Search Strategy

A relevant literature search was conducted using four electronic databases such as PubMed, Scopus, Web of Science, and Google Scholar. The search included terms such as “stroke AND demographic disparities,” “age AND stroke,” gender AND stroke, “ethnicity AND stroke,” “socioeconomic status AND stroke,” “stroke AND cultural and behavioral factors”, and “urban versus rural AND stroke.” The review included articles published in English from 2015 onward to ensure up‐to‐date insights.

### 3.4 Inclusion and Exclusion Criteria

Eligible studies included peer‐reviewed articles and gray literature that examined stroke prevalence or trends in relation to demographic factors. Both quantitative and qualitative studies were considered. Articles were excluded if they did not meet the following criteria: Published outside the 2015–2025 range, not in English, classified as case series, editorials, or conference abstracts, or not directly related to stroke or demographic patterns.

### 3.5 Data Extraction

Data were extracted on key demographic variables (age, sex, race/ethnicity, socioeconomic status, and geographic location), stroke subtypes (ischemic and hemorrhagic), and contributing factors such as healthcare access, comorbidities, and lifestyle‐related risks.

### 3.6 Analysis and Synthesis

The selected studies were thematically analyzed to identify trends, disparities, and contributing factors in stroke prevalence. The synthesis combined quantitative data, such as prevalence rates expressed as percentages across demographic groups, with qualitative insights on social, cultural, and behavioral influences. Differences across demographic and geographic factors were qualitatively summarized.

## 4 Theoretical Frameworks in Public Health and Health Behavior Research

### 4.1 The Health Belief Model

**FIGURE 1:**
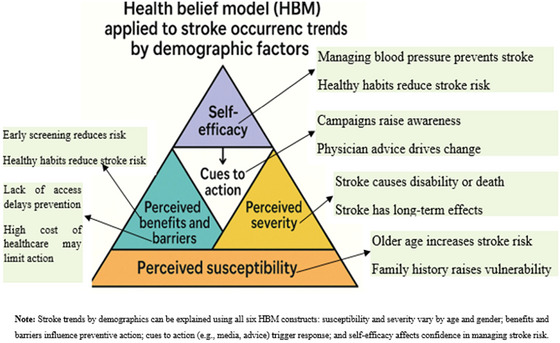
The HBM triangle to explore stroke occurrence trends based on demographic factors.

The Health Belief Model can be effectively applied to understand stroke occurrence trends across different demographic factors (such as age, gender, SES, urban vs. rural, and ethnicity (MJ Alqahtani 2015) (Table 1). When structured as a triangle, each side or step of the model builds upon the others, representing the key components that influence health behavior (Figure 1).

**TABLE 1:** HBM applied to stroke occurrence trends based on demographic factors.

No	HBM constructs	Definition	Perception	Example
1	Perceived susceptibility	Belief about one's risk of stroke (Rountree et al. 2024).	Older adults, post‐menopausal women, African Americans, and low‐SES groups perceive higher stroke risk.	An octogenarian man with hypertension may still think, “It won't happen to him,” unless educated on his specific risk profile.
2	Perceived severity	Belief about how serious stroke is (Oyewole et al. 2020).	Perceptions of stroke severity differ; older adults often see it as life‐threatening.	An elderly person who witnessed stroke‐related disability understands its severity better.
3	Perceived benefits	Belief in the effectiveness of preventive actions (Aycock et al. 2019).	Higher‐educated and wealthier groups value prevention.	A middle‐aged woman understands quitting smoking reduces stroke risk.
4	Perceived barriers	Beliefs about obstacles to prevention (Vincenzo et al. 2022).	Barriers like cost, access, and mistrust hinder prevention, especially in low‐income and rural areas.	A woman finds quitting smoking culturally inappropriate.
5	Cues to action	Factors that trigger preventive action (Tang 2025).	Health messages, and doctor advice prompt action, especially when tailored to cultural contexts.	A church‐based program prompts African Americans to monitor their blood pressure.
6	Self‐efficacy	Confidence in preventing stroke (Nott et al. 2021).	Educated and resource‐rich groups feel more confident.	A young adult adopts a healthy lifestyle, believing they can manage health risks.

### 4.2 The Social Determinants of Health Framework

**FIGURE 2:**
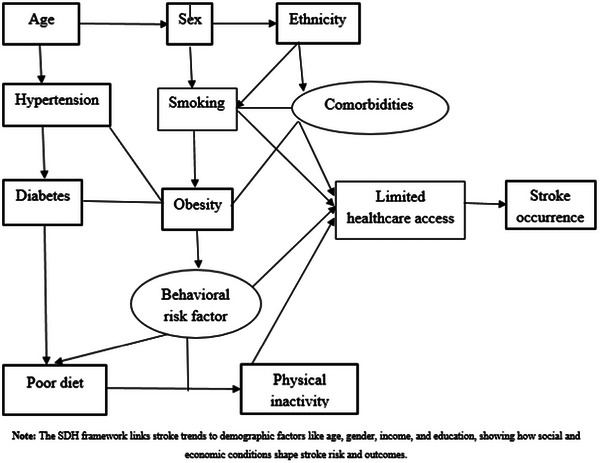
The SDH theory clarifies causal pathways between demographic factors and stroke occurrence.

The Social Determinants of Health (SDH) framework illustrates how broad social conditions contribute to health outcomes such as stroke. It maps out the pathways that clarify why certain demographic groups are at greater risk (Skolarus et al. 2020). For example, upstream factors like unequal access to quality education can limit employment prospects, leading to intermediate conditions such as living in high‐stress areas with poor access to healthy food (Sullivan and Thakur 2020). These daily challenges can result in proximal risk factors like poorly managed hypertension and diabetes, ultimately increasing the likelihood of early stroke with limited access to effective recovery support (Figure 2).

In Figure 2, all causal directions are marked using arrows (▶) and the flow from demographic and environmental factors to stroke risk.

## 5 Results and Discussion

Stroke occurrence is influenced by various demographic factors, leading to significant differences among populations (Avan et al. 2019). These disparities result from a combination of genetics, health behaviors, healthcare access, and SES. The following trends highlight the demographic factors that contribute to the occurrence of stroke.

### 5.1 Age

Stroke prevalence significantly varies with age, with the risk increasing sharply as individuals advance in age (Li et al. 2022). In young adults (under 45 years), strokes are less common but are increasing due to lifestyle factors such as obesity, smoking, diabetes, and illicit drug use (Bukhari et al. 2023). In middle‐aged adults (45–64 years), the risk increases notably due to the prevalence of lifestyle and metabolic conditions, such as hypertension, type 2 diabetes, and dyslipidemia (Xia et al. 2021). In older adults (≥ 65 years), the majority of strokes (approximately 75%) occur in this age group (Li et al. 2022).

Stroke risk is higher in older adults, particularly after 55 years, as aging causes blood vessels to become stiffer and lose elasticity (Rizzoni et al. 2019). This contributes to hypertension and atherosclerosis, which are the major risk factors for stroke. With age, arteries lose their ability to maintain consistent blood flow to the brain, making the brain more vulnerable to ischemic and hemorrhagic stroke. In older populations, damage to small brain blood vessels becomes more frequent, and the brain's ability to compensate for these issues declines, resulting in more severe outcomes during stroke occurrence (Iadecola et al. 2023). Older adults also often have preexisting conditions, such as hypertension, atrial fibrillation, heart failure, coronary artery disease, diabetes, dyslipidemia, and cognitive impairments, which worsen stroke‐related disabilities and complicate recovery. Aging also leads to reduced neuroplasticity, making recovery from stroke more challenging. Over time, poor lifestyle habits such as smoking, unhealthy diet, and lack of physical activity, further increase the risk of stroke in older age (Liu et al. 2023).

Stroke prevalence is rising in younger populations, likely due to lifestyle factors, such as obesity, smoking, and hypertension. The growing prevalence of obesity in younger people contributes to conditions such as hypertension, diabetes, and dyslipidemia, all of which increase the risk of stroke (Avan et al. 2019). Tobacco use and excessive alcohol consumption, which are common among younger adults, are the major risk factors for stroke. Lack of physical activity contributes to obesity and poor cardiovascular health, both of which are associated with stroke. In addition, certain genetic or acquired conditions such as antiphospholipid syndrome or sickle cell disease can increase the risk of ischemic stroke. The use of illicit drugs, such as cocaine, amphetamines, and other recreational substances, can cause vasospasm, vascular injury, and embolism, leading to stroke in younger adults. Conditions such as lupus, vasculitis, patent foramen oval, infective endocarditis, and cervical artery dissection can cause vascular inflammation, further increasing the risk of stroke (Bukhari et al. 2023). Migraines with aura are associated with an increased risk of stroke, particularly in women who smoke or use estrogen‐containing contraceptives. Some oral contraceptives can increase clot formation, particularly when combined with other risk factors. The role of hormone replacement therapy (HRT) in stroke risk is debated, with some forms of HRT potentially increasing the risk depending on the type of hormones, dosage, and timing of initiation (Iadecola et al. 2023).

**FIGURE 3:**
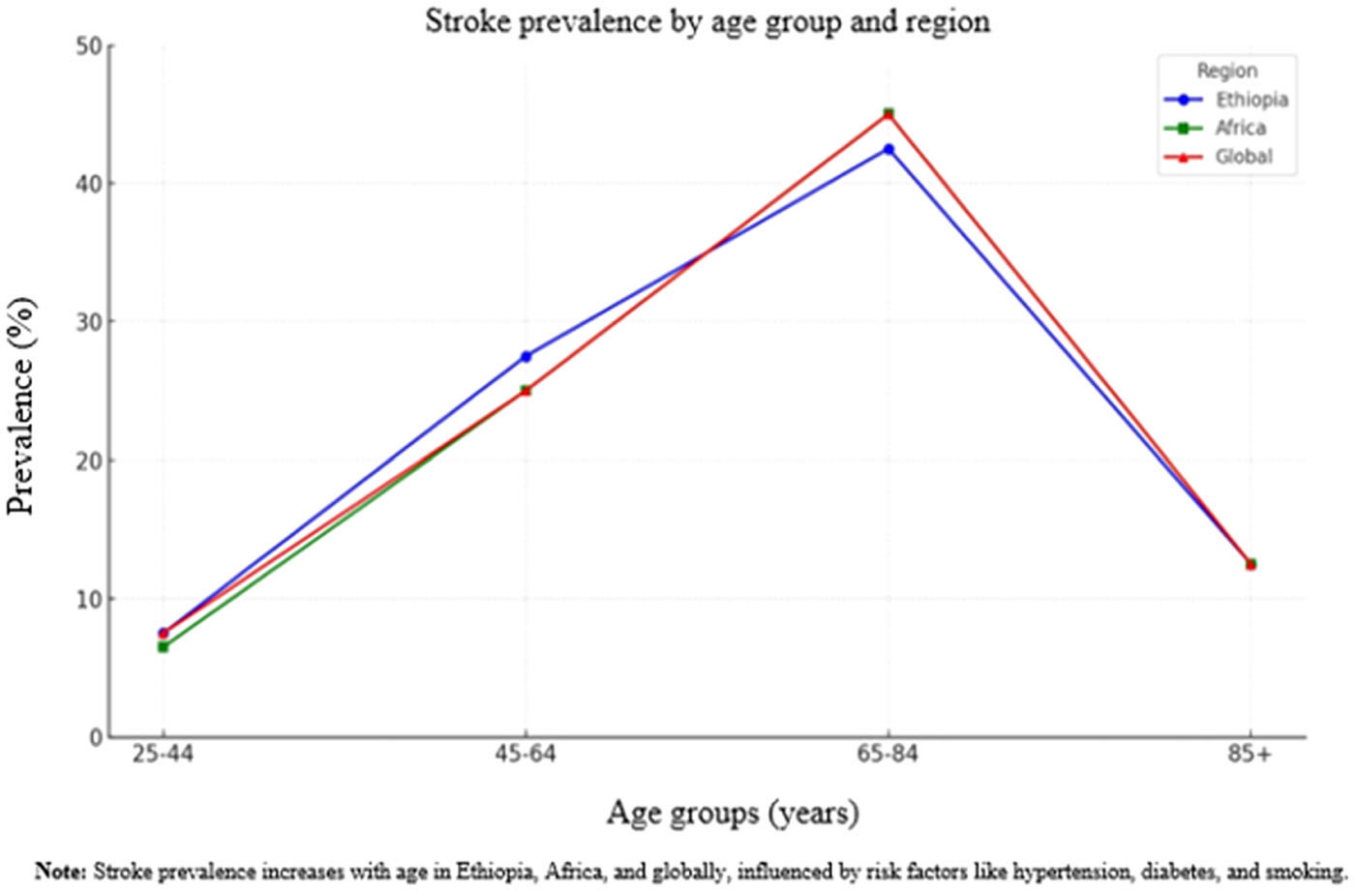
The prevalence of stroke in different age groups across different regions.

Here is an overview of stroke prevalence by age group, including the age ranges of 25–44, 45–64, 65–84, and 85 years and older, with specific assumptions and comparisons across different regions (Figure 3).

#### 5.1.1 Ethiopia

In Ethiopia, stroke is uncommon in the 25–44 years age group but is becoming more frequent due to increasing risk factors such as hypertension and smoking. This group is estimated to represent 5%–10% of stroke cases, with men being more affected. In the 45–64 years age group, the prevalence of stroke increased, with men being more affected (Misgana et al. 2023). This group accounts for approximately 25%–30% of all stroke cases as risk factors, such as hypertension, diabetes, and smoking, become more prevalent. Stroke prevalence peaks in the 65–84 years age group, contributing to 40%–45% of stroke cases. Age‐related factors such as arterial stiffening, chronic conditions, and hormonal changes increased the risk in this group. In those aged 85 years and older, stroke prevalence continues to rise, representing 10%–15% of all stroke cases, with frailty and multiple comorbidities increasing the risk of stroke (Abate et al. 2021).

#### 5.1.2 Africa

In the 25–44‐year‐old age group across Africa, stroke is rare, accounting for 5%–8% of cases, although its prevalence is rising due to higher hypertension rates and lifestyle changes. For the 45–64 years age group, stroke prevalence increases, accounting for about 20%–30% of stroke cases in Africa (Akinyemi et al. 2021). Risk factors, such as hypertension, diabetes, and urbanization, contribute to a higher prevalence. Stroke prevalence significantly rises in the 65–84 years age group, which represents 40%–50% of all strokes. Aging and the growing prevalence of chronic diseases contribute to a higher incidence of stroke. In individuals aged ≥ 85 years, stroke prevalence remains high, accounting for 10%–15% of stroke cases, driven by the presence of multiple chronic conditions (Adeloye 2014).

#### 5.1.3 Global

Globally, the stroke prevalence is relatively low in the 25–44 years age group, representing 5%–10% of all cases. This group is more likely to be affected, with early onset strokes often linked to genetic factors and lifestyle choices such as smoking and high cholesterol levels. In the 45–64 years age group, stroke prevalence increased, accounting for 20%–30% of all cases globally (Feigin et al. 2022). This increase is attributed to higher rates of hypertension, diabetes, and obesity. Stroke is most prevalent in the 65–84 years age group, contributing to 40%–50% of global stroke cases. Aging‐related changes and chronic conditions such as atrial fibrillation and diabetes are significant risk factors. For individuals aged ≥ 85 years, stroke prevalence remains high, representing 10%–15% of global stroke cases (Kim et al. 2020).

Figure 3 shows that stroke prevalence in Ethiopia (blue) increases with age, peaking in the 65–84 age group. However, it significantly decreases in the 85+ age group, even though this group is highly experienced stroke. Africa (green), there is an upward trend, though stroke prevalence is slightly lower than that in Ethiopia for most age groups. Globally (red), stroke prevalence is similar to that in Africa, but slightly higher in the 25–44 age group. The stroke prevalence trends for Africa and the global figures overlap in all age categories, except for the 25–44 age group. For the 45–64 and 65–84 age groups, Ethiopia's prevalence (blue) shows a slight divergence, but the overall trends remain quite similar to those of Africa and the global average.

### 5.2 Gender

Sex significantly influences stroke risk, and outcomes, with differences attributed mainly to biological, hormonal, and social factors. In women, estrogen provides protective effects on blood vessels, reducing stroke risk during the reproductive years (Lagranha et al. 2018). However, after menopause, the decline in estrogen levels increases the risk of stroke. Some studies indicate that HRT and certain oral contraceptives may increase stroke risk, particularly in women who smoke or have other risk factors such as high blood pressure (Johansson et al. 2022). Pregnancy‐related conditions such as preeclampsia, eclampsia, and gestational diabetes also increase stroke risk, especially during the postpartum period, when changes in blood clotting and circulation heighten vulnerability (Miller and Leffert 2020). Women with hypertension, atrial fibrillation, psychosocial stress, and depression have an elevated risk of stroke. Gender differences in smoking and alcohol consumption also impact stroke risk, with men often exhibiting higher levels of these behaviors. Premenopausal women benefit from the protective effects of estrogen, which helps maintain vascular health, but this advantage decreases after menopause, leading to a higher stroke risk in older women (Naftolin et al. 2019).

In men, testosterone levels are associated with higher blood pressure and cholesterol levels, both of which are major risk factors for stroke. Men are more likely to engage in smoking, excessive alcohol consumption, and poor dietary habits, all of which contribute to stroke risk (Akpalu et al. 2019). In addition, conditions such as obesity, hypertension, and diabetes are often less well‐managed in men, further increasing the risk of stroke. Men are also less likely to visit physicians regularly, which may result in delayed diagnosis and treatment of risk factors such as hypertension and diabetes (Powell et al. 2019). Men experience and manage stress differently, contributing to a higher risk of cardiovascular and cerebrovascular events. Some studies have suggested that men may have less efficient collateral circulation in the brain, which could lead to more severe stroke outcomes. Genetic factors related to clotting and vascular health may also predispose men to stroke more than women (Boehme et al. 2017).

Postmenopausal women face an increased stroke risk compared to premenopausal women owing to various physiological, hormonal, and lifestyle changes (Prabakaran et al. 2021). The decline in estrogen levels after menopause eliminates its protective effects on blood vessels and lipid profiles (Nie et al. 2022). Postmenopausal women are more likely to experience conditions such as vascular stiffness, endothelial dysfunction, hypertension, dyslipidemia (higher LDL and lower HDL cholesterol), diabetes, weight gain, and central obesity, all of which increase the risk of stroke. Lifestyle factors such as a sedentary lifestyle, smoking, and poor diet further exacerbate this risk (Park et al. 2020).

**FIGURE 4:**
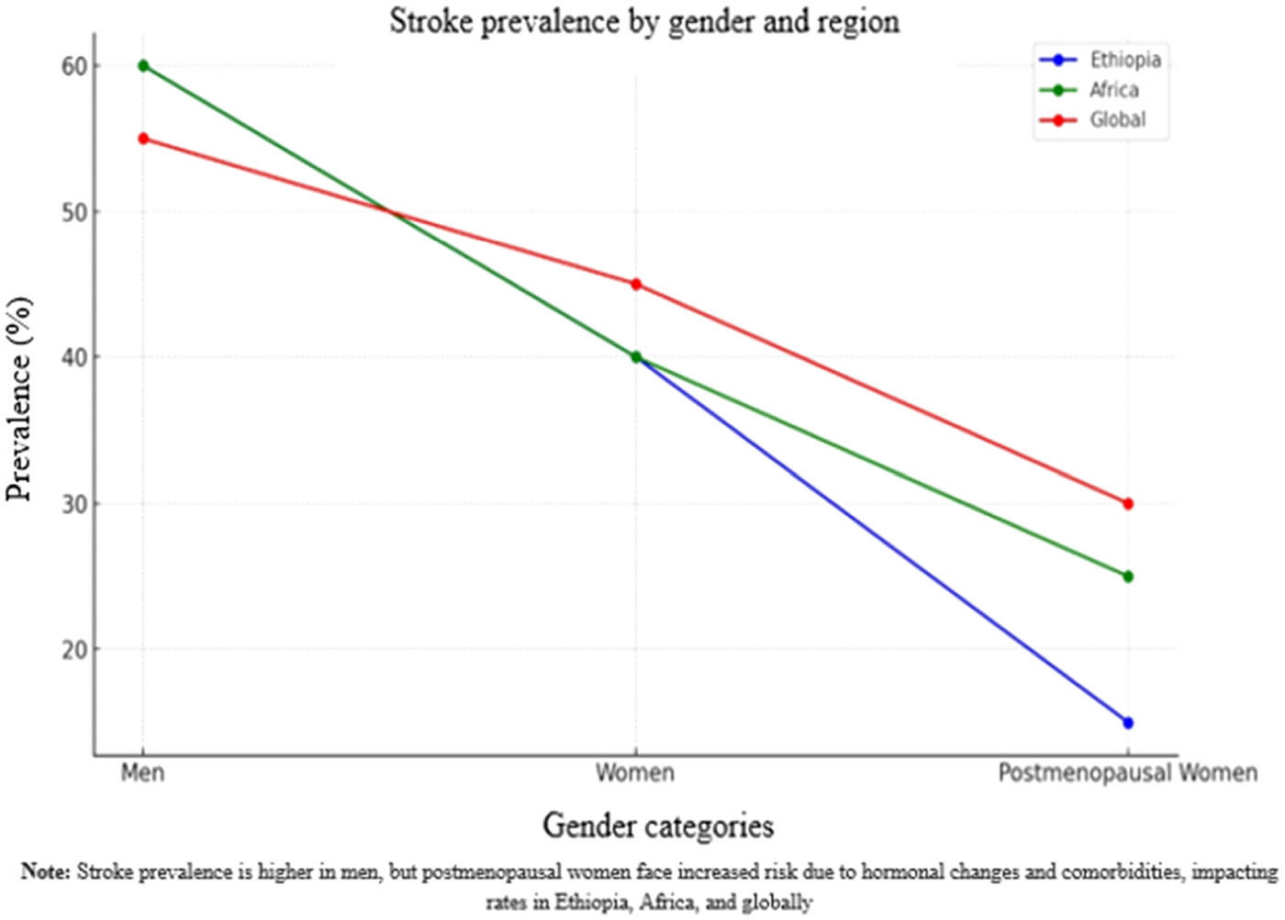
The prevalence of stroke in different genders across regions.

Here, is an overview of stroke prevalence based on sex in Ethiopia, Africa, and globally, with specific assumptions to compare men, women, and postmenopausal women (Figure 4).

#### 5.2.1 Ethiopia

Stroke is a leading cause of morbidity and mortality in Ethiopia, with a noticeable increase in prevalence over the past decade. Some studies have shown that men are more affected by stroke than women, particularly in urban areas. However, this sex gap narrows among postmenopausal women due to hormonal changes that influence stroke risk. Some studies found that men account for about 60%–65% of total stroke cases and experience higher mortality rates than women (Sahle Adeba et al. 2022). Conversely, although women have a lower initial stroke prevalence, they face a greater risk of stroke‐related death. Some studies have shown that stroke contributes to 35%–40% of female mortality in Ethiopia. Among postmenopausal women, stroke prevalence rises by 10%–15% compared to that in premenopausal women (Abdu et al. 2022).

#### 5.2.2 Africa

In Africa, stroke is increasingly recognized as a major health issue, with varying prevalence across regions. A systematic review and meta‐analysis reported a prevalence of 2%–5% in different African countries, with men, especially middle‐aged adults, at higher risk (Fowobaje et al. 2024). However, the risk in women significantly increases after menopause. Men in Africa account for 55%–60% of stroke cases, with hypertension being a major contributing factor. For women, particularly those over 55 years, the risk of stroke increases, representing 40%–45% of all stroke cases, with hypertension and diabetes becoming a growing concern (Akinkugbe 1976). Some studies highlighted that postmenopausal women have a 20%–25% higher prevalence of stroke than their premenopausal counterparts due to hormonal shifts and the presence of comorbidities such as hypertension and diabetes (Okonkwo et al. 2022).

#### 5.2.3 Global

Globally, stroke is a major cause of death and disability, with an estimated 15 million people suffering from stroke annually. Men tend to experience stroke at younger ages, leading to higher incidence rates in earlier life stages, while the incidence becomes more evenly distributed in older age groups. Men account for 55%–60% of stroke cases worldwide, while women represent 40%–45%, with the risk for women increasing with age, especially owing to hormonal changes, hypertension, and diabetes (Li et al. 2022). Some studies have shown that postmenopausal women face a significantly higher stroke risk, with their likelihood of stroke being 15%–30% higher than that of premenopausal women, primarily due to the loss of estrogen's protective effects (Demel et al. 2018).

In Figure 4, the blue line represents Ethiopia, where men have a higher prevalence of stroke than women, with a sharp decline observed in postmenopausal women. The green line, representing Africa, also shows a higher stroke prevalence among men than women, but the decline in postmenopausal women is more gradual compared to Ethiopia. The red line, representing global trends, indicates that men have a higher stroke prevalence globally than women, with a moderate decline in postmenopausal women. The prevalence trends for women and postmenopausal women in Africa (green) and globally (red) are somewhat similar, showing significant overlap. In contrast, Ethiopia's prevalence trends (blue) for men and postmenopausal women are more distinct. However, it has begun to align more closely with the trends observed among women in Africa and globally.

### 5.3 Race

Race and ethnicity significantly affect stroke risk and prevalence, with various factors related to genetics, SES, healthcare access, and lifestyle choices. Black and Hispanic/Latino populations: The high prevalence of stroke in these groups is influenced by a mix of biological, cultural, and healthcare‐related factors. Conditions such as sickle cell disease increase the risk of ischemic stroke, particularly in children (Mehta et al. 2023). These populations often face challenges with access to health insurance, which limits both preventive care and timely treatment. Socioeconomic disadvantages such as reduced access to healthy food, exercise, and healthcare further increase the risk of stroke. High‐stress levels due to systemic racism may also contribute to unhealthy dietary habits and lower physical activity levels. Genetic factors, including predisposition to hypertension, obesity, and diabetes, play a role in increasing stroke risk (Huang 2024).

**Asian populations**: Stroke prevalence is notably high among some Asian populations owing to a combination of dietary, lifestyle, and genetic factors. Although some Asian diets are rich in vegetables and fish, they may contain excessive sodium and insufficient potassium, contributing to stroke risk. High smoking rates in certain regions and alcohol consumption are risk factors. Genetic predispositions such as higher susceptibility to hypertension, diabetes, and clotting disorders can increase the risk of stroke (Boehme et al. 2017). In addition, limited access to early diagnosis and treatment may result in a higher incidence of stroke and poorer outcomes.

**African Americans**: African Americans have nearly double the risk of first stroke compared to Caucasians. This higher risk is linked to prevalent conditions, such as hypertension, diabetes, obesity, and sickle cell disease. Sickle cell disease, which is more common among African Americans, increases the risk of ischemic stroke, particularly in younger individuals. Factors such as a low SES, inadequate healthcare access, and limited insurance coverage can delay diagnosis and treatment, further increasing the risk of stroke. Unhealthy diets, lack of physical activity, and higher rates of smoking and alcohol use exacerbate this risk (Carnethon et al. 2017).

**Caucasians**: However, Caucasians have a lower overall stroke prevalence, they are more likely to experience large‐artery atherosclerosis or cardioembolic stroke. Atherosclerosis, a major factor in ischemic stroke, is more common in this group and is influenced by conditions such as high cholesterol levels, atrial fibrillation, and a family history of cardiovascular disease. Stroke risk increases with age, and because Caucasians generally live longer, the prevalence of stroke is higher among older individuals. Poor nutrition and smoking, especially among middle‐aged and older adults, also significantly contribute to stroke risk (Wang et al. 2022).

**Native Americans/Alaska Natives**: Native American and Alaska Native populations have some of the highest stroke prevalence, particularly ischemic strokes. These populations have high rates of hypertension, diabetes, and obesity, which are key risk factors for stroke. Limited access to healthcare exacerbates the impact of stroke by hindering timely diagnosis and treatment (Boden‐Albala et al. 2017).

**Middle Eastern and North African populations**: Although stroke prevalence in Middle Eastern and North African populations is generally lower than those in African Americans, they remain comparable to other ethnic groups. Hypertension is a major risk factor, and rising rates of diabetes and obesity, driven by lifestyle changes and the adoption of Western diets, are increasing the stroke risk in these populations (Fleischer and Sadek 2024).

**FIGURE 5:**
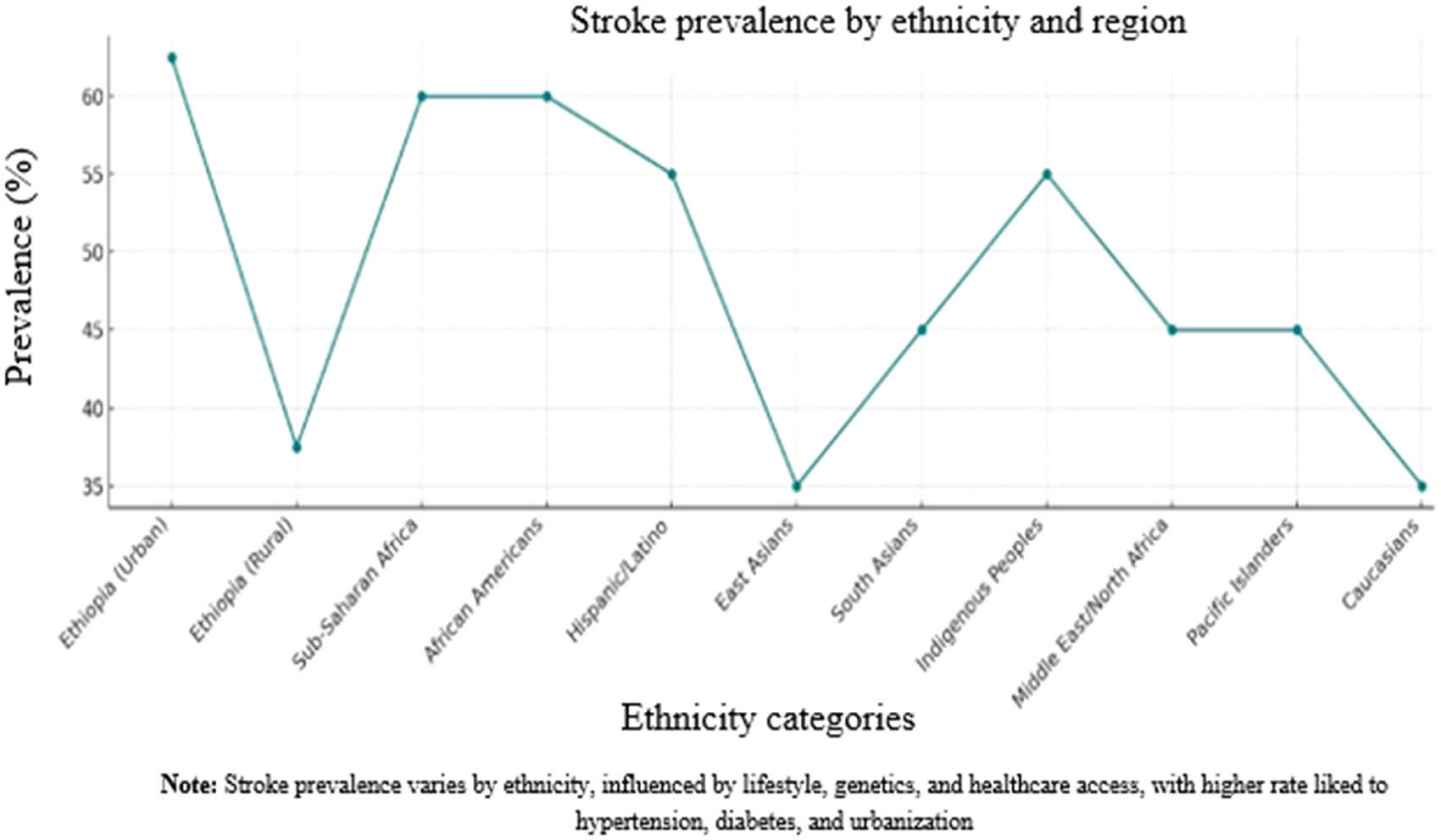
The prevalence of stroke in different ethnicities across regions.

Here is an overview of stroke prevalence based on ethnicity in Ethiopia, Africa, and globally with specific assumptions (Figure 5). The prevalence of stroke varies across ethnicities owing to different genetic, environmental, and lifestyle factors.

#### 5.3.1 Ethiopia

Stroke is a growing health issue in Ethiopia, but comprehensive data on its ethnicity‐based prevalence are limited. Ethnic groups in Ethiopia, such as Oromo, Amhara, and Tigray, may experience different stroke prevalence, which is largely influenced by socioeconomic and geographical factors (Baye et al. 2020). Rural areas show lower stroke prevalence than urban regions, as urban areas tend to have higher rates of hypertension, diabetes, and lifestyle‐related factors that increase stroke risk. In urban areas, there is a higher prevalence of stroke due to better diagnosis and lifestyle‐related risk factors, contributing to approximately 60%–65% of stroke cases. In rural areas, stroke prevalence is lower, but increases due to the rise of hypertension and diabetes, accounting for 35%–40% of stroke cases (Gidey and Hailu 2023).

#### 5.3.2 Africa

The prevalence of stroke in sub‐Saharan Africa has been increasing, with urbanization and lifestyle changes contributing to higher stroke rates. However, rural areas still have a lower reported stroke prevalence, partly because of underreporting and limited access to healthcare. Sub‐Saharan Africans have a high stroke prevalence, with some studies suggesting that the prevalence of stroke is approximately 50%–70% higher in African populations than in global averages (Abissegue et al. 2024). Hypertension is the leading risk factor, along with other contributing factors, including obesity, smoking, and limited access to healthcare.

#### 5.3.3 Global

**African Descent (African American, Sub‐Saharan Africans)**: In African Americans, stroke rates are 2–3 times higher than the Caucasians in the United States (Carnethon et al. 2017). Stroke is one of the leading causes of death among African Americans, with significant risk factors, such as hypertension, diabetes, and genetic predisposition (Akinyemi et al. 2021). In sub‐Saharan Africa, the prevalence of stroke is rising rapidly, with studies showing a high prevalence of stroke, especially in urban areas. Rates of stroke‐related mortality are higher in sub‐Saharan Africa due to limited access to healthcare (Mohammed et al. 2021). Stroke accounts for approximately 50%–60% of all stroke cases in both African Americans and sub‐Saharan Africans (Akinyemi et al. 2021).

Hispanic/Latino descent has a higher stroke prevalence than non‐Hispanic Caucasians, with significant disparities in stroke outcomes, particularly in the United States. Risk factors such as hypertension, diabetes, and obesity contribute to high rates of stroke in this group (Gardener et al. 2020). Stroke prevalence in Hispanic/Latino populations is estimated to be approximately 50%–60% higher than that in the general population in the United States and Latin America, largely driven by hypertension and obesity (Shaw et al. 2018).

In East Asian Descent (Chinese, Japanese, and Koreans), stroke is a leading cause of death in East Asia, particularly in China and Japan. The stroke rate is generally lower in East Asia than in Africa and the Americas; however, it remains a significant public health concern. Stroke prevalence in East Asia accounts for approximately 30%–40% of stroke cases in this region, with a higher burden of ischemic stroke in countries like China (Venketasubramanian et al. 2017).

South Asians (Indian, Pakistani, Sri Lankan) have a higher stroke prevalence, particularly ischemic stroke, due to a combination of genetic and environmental factors, such as high rates of diabetes, hypertension, and metabolic syndrome (Zhang et al. 2023). Studies suggest that the stroke burden in South Asia is rising owing to increasing rates of urbanization, poor dietary habits, and lack of awareness. Stroke prevalence in South Asia is approximately 40%–50% higher than that in other populations due to these risk factors (Singh et al. 2017)

Indigenous populations (Native Americans and Aboriginal Australians) experience higher rates of stroke than the general population. The higher prevalence is due to the greater burden of comorbidities, such as hypertension, diabetes, and smoking.

Stroke prevalence in indigenous populations is estimated to be approximately 50%–60% higher, with poorer outcomes due to delayed healthcare access and high comorbidities (Gardiner et al. 2021).

In Middle Eastern and North African Descent, stroke is a rising burden in countries such as Egypt, Saudi Arabia, and Iran, which is attributed to urbanization, changes in diet, and an aging population (Jaberinezhad et al. 2022). Stroke prevalence in the Middle Eastern and North African populations is increasing, with estimates suggesting that 40%–50% of stroke cases in these regions are related to lifestyle factors, such as high salt intake, diabetes, and hypertension (Shahbandi et al. 2022).

Pacific Islander Descent (Hawaiians and Fijians) has a higher prevalence of stroke, largely due to lifestyle factors, such as obesity, diabetes, and high‐fat diets. Stroke prevalence in Pacific Islanders is estimated to be 40%–50% higher than that in other populations owing to these risk factors (Venketasubramanian 2021).

In Caucasians (European descent), stroke rates are moderate compared to those in other ethnicities, but higher than those in East Asia. The stroke burden in Europe and the United States is largely driven by hypertension, atrial fibrillation, and smoking, although healthcare systems have helped to reduce stroke mortality rates. Stroke prevalence in Caucasians is approximately 30%–40% of stroke cases globally, with a higher prevalence in Eastern Europe and Russia due to lifestyle factors (Yesilot et al. 2017).

In Figure 5, the blue line representing Ethiopia indicates a significantly higher prevalence of stroke in urban areas compared to rural areas. Ethiopia's data also reveal more distinct patterns between urban and rural populations, with trends that often diverge from global averages. In addition, the prevalence of stroke varies significantly among different ethnic groups. Globally, populations of sub‐Saharan African and African American descent tend to have the highest prevalence of stroke. Hispanic/Latino and indigenous populations also tend to have higher prevalence rates than those of Caucasian or Pacific Islander descent. Some overlap exists between ethnic categories, such as sub‐Saharan African and African American, as well as between Hispanic/Latino and indigenous groups. Additional overlap can also occur among ethnicities of Middle Eastern or North African, South Asian, and Pacific Islander descent.

### 5.4 Socioeconomic Status

SES can have a significant impact on stroke risk, particularly in lower‐income groups, who are more vulnerable due to various factors, such as SDH, lifestyle habits, and limited healthcare access (Marshall et al. 2015). In urban areas, factors such as air pollution and high stress levels contribute to an increased risk of stroke as both are linked to cardiovascular diseases. Urban residents may also have easier access to unhealthy food, alcohol, and tobacco, all of which increase the risk of stroke (Kapral et al. 2019). In addition, people in cities often face socioeconomic struggles such as poverty and high living costs, which can negatively affect their health, including increasing the likelihood of stroke (Misgana et al. 2023). Mental health issues, such as depression and anxiety, are more common in urban environments, which can also increase the risk of stroke and complicate recovery. The scarcity of green spaces and the prevalence of heavy traffic reduce opportunities for physical activity, which is crucial for preventing stroke. Lack of exercise increases the risk of developing conditions such as hypertension, diabetes, and obesity, which, in turn, contributes to a higher risk of stroke (Prabakaran et al. 2021).

In rural areas, the absence of specialized healthcare facilities, such as stroke units, and lack of access to neurologists or stroke experts may delay diagnosis and treatment. Emergency medical services may take longer to reach rural areas, further delaying life‐saving interventions such as clot‐busting medications or surgery (Pandian et al. 2020). Lower SES in rural populations is often associated with lower health literacy, meaning that people may not be as informed about stroke prevention, symptoms, or the importance of seeking early medical care, resulting in treatment delays (Chen et al. 2021). While rural residents may engage in more physical activity through farming or outdoor work, they often face challenges related to limited access to healthy food, contributing to higher rates of obesity, smoking, and poor diet, which are all risk factors for stroke (Kris‐Etherton et al. 2020). Though air quality might be better in rural areas, limited access to preventive care and health education can leave cardiovascular risks unaddressed (Thompson et al. 2019). In addition, rural populations often experience higher rates of conditions, such as hypertension, diabetes, and smoking, which increase the risk of stroke. The living conditions of people with lower SES backgrounds, including overcrowded housing, poor air quality, and limited access to parks or recreational spaces, further exacerbate health disparities and increase the likelihood of stroke (Pantoja‐Ruiz et al. 2025).

**FIGURE 6:**
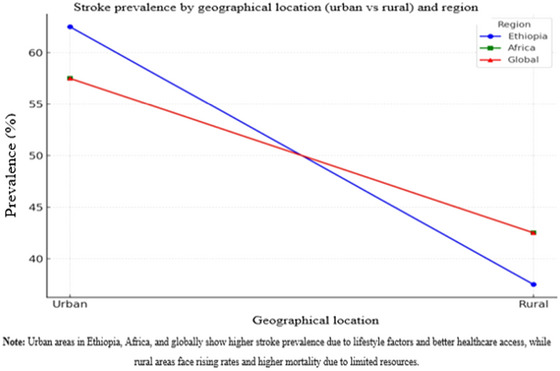
The prevalence of stroke in different geographical locations across regions.

The prevalence of stroke differs considerably between urban and rural areas owing to variations in healthcare access, lifestyle choices, and socioeconomic factors. Below is a summary of stroke prevalence in urban and rural areas in Ethiopia, Africa, and globally, with specific assumptions (Figure 6).

#### 5.4.1 Ethiopia

Stroke prevalence tends to be higher in urban areas of Ethiopia, primarily due to improved access to healthcare, a greater prevalence of risk factors such as hypertension, smoking, sedentary lifestyles, and better diagnostic facilities. Some studies suggest that urban regions account for 60%–65% of stroke cases, as urban populations generally exhibit better health‐seeking behaviors and have more diagnosed cases (Melak et al. 2021). In contrast, rural areas have a lower stroke prevalence, although the burden is rising owing to changing lifestyles. However, underreporting and limited healthcare access means that fewer cases are diagnosed. Rural areas represent approximately 35%–40% of stroke cases, with higher mortality rates due to delayed care (Alene et al. 2020).

#### 5.4.2 Africa

In Africa, urban areas have a higher stroke prevalence owing to factors such as higher rates of hypertension, diabetes, obesity, and smoking. Some studies show that urban populations contribute to 55%–60% of stroke cases, driven by modern lifestyle risks and better healthcare access (Peer et al. 2021). Although stroke rates are lower in rural Africa, they are on the rise due to demographic shifts, such as aging populations, and increasing risk factors such as hypertension. Rural areas contribute to 40%–45% of stroke cases, with higher mortality due to limited treatment access and delays in seeking care (Smythe et al. 2022).

#### 5.4.3 Global

Worldwide, urban areas have a higher stroke prevalence, influenced by lifestyle factors, such as sedentary behavior, smoking, alcohol consumption, and greater healthcare access. It is estimated that 55%–60% of stroke cases occur in urban areas, especially in high‐income countries with well‐established healthcare systems (Feigin et al. 2023). In rural areas globally, stroke rates are lower but rising due to the growing prevalence of risk factors, such as hypertension, coupled with delayed healthcare access, leading to higher mortality rates. Rural areas contribute to 40%–45% of stroke cases, but with greater mortality compared to urban populations (Kapral et al. 2019).

In Figure 6, the blue line (representing Ethiopia) shows that stroke prevalence in urban areas is the highest among all regions, ranging from 60% to 65%. The green line (representing Africa) indicates a slightly lower stroke prevalence in urban areas, at 55%–60%, while the red line (representing the global trend) reflects a similar pattern to Africa, with stroke prevalence in urban areas also at 55%–60%. This overlap between the green and red lines suggests an identical stroke prevalence in urban and rural areas across Africa and globally. In rural areas, the green and red lines are completely overlapped, while the blue line converges with or nearly overlaps the green or red lines within the 40%–45% range, indicating similar stroke prevalence trends in Ethiopia, Africa, and globally.

### 5.5 Cultural and Behavioral Factors

Cultural and behavioral factors play a crucial role in the risk of stroke, affecting its prevalence, symptoms, and outcomes through lifestyle choices, health behaviors, and social conditions (Kandula et al. 2019). Diets that are low in fruits, vegetables, and whole grains can lead to higher cholesterol levels and blood pressure, both of which increase the risk of stroke. In some cultures, sedentary lifestyles are more common owing to social norms, work habits, or economic challenges, which can lead to obesity, hypertension, and diabetes conditions linked to stroke (Piano 2017). Smoking and alcohol consumption together have a great effect on stroke risk as they negatively impact blood pressure, clotting, and heart health (Brewin et al. 2020). In addition, some individuals may delay seeking medical care because of mistrust of the healthcare system or adherence to traditional health beliefs, which can worsen stroke outcomes. Chronic stress and mental health challenges, influenced by cultural expectations and societal pressures, also elevate risks. Certain groups may also have genetic predispositions such as sickle cell disease, which increases the likelihood of stroke (Ng et al. 2020).

**FIGURE 7:**
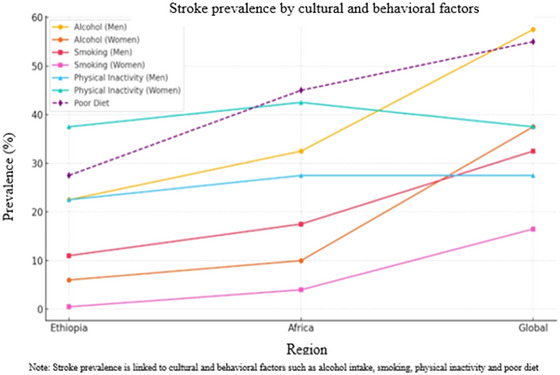
The prevalence of stroke in different cultural and behavioral factors across regions.

Alcohol consumption, smoking, a sedentary lifestyle, and poor diet are significant contributors to chronic diseases, such as cardiovascular disease, stroke, and diabetes (Piano 2017). These behaviors are influenced by region, gender, and SES. Below is a comparison of these health risk factors based on specific assumptions for Ethiopia, Africa, and globally (Figure 7).

#### 5.5.1 Ethiopia

Alcohol consumption in Ethiopia is relatively low compared with that in other regions. Some studies indicate that 12%–15% of adults drink alcohol regularly, with higher rates among men (Ayano et al. 2019). Around 20%–25% of men consume alcohol regularly, while only 5%–7% of women do so. In terms of smoking, approximately 5%–8% of Ethiopian adults smoke, with higher rates in urban areas. Smoking is predominantly a male habit, with 10%–12% of men smoking compared to less than 1% of women (Handebo et al. 2020). Regarding physical inactivity, 30%–35% of adults are physically inactive, with a higher percentage of women leading sedentary lifestyles. About 35%–40% of women are physically inactive, while 20%–25% of men are physically inactive (Abdeta et al. 2018). Poor dietary habits are increasing, with 25%–30% of Ethiopians consuming unhealthy diets, especially in urban areas. Both men and women have similar poor dietary patterns, although women are more likely to suffer from micronutrient deficiencies (Seyoum Keflie et al. 2018).

#### 5.5.2 Africa

Alcohol consumption in Africa varies by country, with 20%–30% of adults reporting regular drinking (Vellios and Van Walbeek 2018). Men account for most of the alcohol consumption, with 30%–35% of men drinking regularly compared to 8%–12% of women. Smoking rates in Africa are generally lower, with about 10%–12% of adults smoking. Smoking is more common among men, with 15%–20% of men smoking compared to 3%–5% of women. Physical inactivity is becoming more prevalent, particularly in urbanized regions. Around 30%–40% of African adults are physically inactive, with a higher rate among women, where 40%–45% of women are sedentary compared to 25%–30% of men (Boehme et al. 2017). A poor diet is also common, with 40%–50% of African adults having unhealthy eating habits, leading to increased obesity rates and related diseases (Elmberg Sjöholm et al. 2021).

#### 5.5.3 Global

Alcohol consumption is more widespread, with 45%–50% of adults reporting alcohol use within the past year (Boot et al. 2020). The prevalence is even higher in high‐income countries, often exceeding 60%. About 55%–60% of men and 35%–40% of women worldwide regularly consume alcohol. Smoking is more common globally, with 20%–25% of adults reporting smoking (Abeysekera et al. 2024). In high‐income countries, smoking is more common among men, with 30%–35% of men and 15%–18% of women smoking. In lower‐income regions, there is a smaller gender gap in the smoking rates. Globally, 30%–40% of adults do not meet the recommended physical activity levels, and women are more likely to be inactive, with 35%–40% of women compared to 25%–30% of men (Salmantabar et al. 2022). A poor diet is a significant issue worldwide, with 50%–60% of adults consuming unhealthy diets, leading to obesity and chronic diseases (Al‐Jawaldeh and Abbass 2022). This is characterized by a high intake of sugar, salt, and fats and insufficient consumption of fruits and vegetables.

Figure 7 illustrates the prevalence of alcohol consumption, smoking, physical inactivity, and poor dietary habits in Ethiopia, Africa, and globally, categorized by sex. Alcohol consumption, smoking, and physical inactivity are depicted in straight lines, emphasizing their significant roles in stroke prevalence. Poor diet is depicted with a dashed line, also highlighting its substantial contribution to stroke risk. Ethiopia reported the lowest prevalence for most factors, except for physical inactivity among women, while global prevalence was consistently the highest. Poor diets show the sharpest global increase, reflecting the rising consumption of processed and unhealthy foods. Men tend to have higher rates of alcohol use and smoking, whereas women face greater challenges with physical inactivity and dietary issues, including micronutrient deficiencies. The overlaps in the graph emphasize the interconnection of behavioral risk factors, particularly in Africa and at the global level.

### 5.6 Strengths and Limitations

This integrative review synthesizes the literature on stroke prevalence, emphasizing demographic disparities. By incorporating findings from diverse populations and geographic regions, including both qualitative and quantitative studies, it provides insights into how social and structural determinants influence stroke occurrence. However, reliance on secondary data may introduce publication bias, especially due to the underrepresentation of marginalized groups in primary studies. The review excludes children and late adolescents, focusing on young adults, middle‐aged individuals, and older adults, meaning the findings may not reflect the stroke burden in those aged 24 or younger, who may have different risk factors and causes. In addition, the lack of formal quality appraisal of the included studies may compromise the strength and reliability of the conclusions.

### 5.7 Future Directions

Future research should focus on, integrating SDH, such as education, occupation, and cultural practices, into stroke research to also explore the effectiveness of targeted interventions and health policies aimed at reducing disparities in stroke outcomes across different demographic and geographic groups.

## 6 Conclusions

Demographic disparities in stroke occurrence result from complex interactions between biological, social, and environmental factors. These differences are shaped by factors such as age, sex, ethnicity, SES, geographic location, and access to healthcare. Addressing these disparities requires multifaceted strategies including policy reforms, targeted public health programs, and healthcare interventions that prioritize equity.

## Author Contributions

**Gudisa Bereda**: data curation, conceptualization, methodology, software, investigation, validation, formal analysis, visualization, project administration, resources, writing – original draft, writing – review and editing.

## Conflicts of Interest

The author declares no conflicts of interest.

## Peer Review

The peer review history for this article is available at https://publons.com/publon/10.1002/brb3.70575

## Data Availability

Data sharing is not applicable to this article as no new data were created or analyzed in this study.

## References

[brb370575-bib-0001] Abate, T. W. , B. Zeleke , A. Genanew , and B. W. Abate . 2021. “The Burden of Stroke and Modifiable Risk Factors in Ethiopia: A Systemic Review and Meta‐Analysis.” PLoS ONE 16, no. 11: e0259244. 34723996 10.1371/journal.pone.0259244 PMC8559958

[brb370575-bib-0002] Abdeta, C. , Z. Teklemariam , and B. Seyoum . 2018. “Prevalence of Physical Inactivity and Associated Factors Among Adults in Harar Town, Eastern Ethiopia.” Baltic Journal of Health and Physical Activity 10, no. 2: 72–80.

[brb370575-bib-0003] Abdu, H. , and G. Seyoum,. 2022. “Sex Differences in Stroke Risk Factors, Clinical Profiles, and in‐Hospital Outcomes Among Stroke Patients Admitted to the Medical Ward of Dessie Comprehensive Specialized Hospital, Northeast Ethiopia.” Degenerative Neurological and Neuromuscular Disease 12: 133–144. 36304698 10.2147/DNND.S383564 PMC9595065

[brb370575-bib-0004] Abeysekera, I. , R. De Silva , D. Silva , L. Piumika , R. Jayathilaka , and L. Rajamanthri . 2024. “Examining the Influence of Global Smoking Prevalence on Stroke Mortality: Insights From 27 Countries Across Income Strata.” BMC Public Health 24, no. 1: 857. 38504226 10.1186/s12889-024-18250-1 PMC10953178

[brb370575-bib-0005] Abissegue, G. , S. I. Yakubu , A. S. Ajay , and F. Niyi‐Odumosu . 2024. “A Systematic Review of the Epidemiology and the Public Health Implications of Stroke in Sub‐Saharan Africa.” Journal of Stroke and Cerebrovascular Diseases 33, no. 8: 107733. 38663647 10.1016/j.jstrokecerebrovasdis.2024.107733

[brb370575-bib-0006] Adeloye, D. 2014. “An Estimate of the Incidence and Prevalence of Stroke in Africa: A Systematic Review and Meta‐Analysis.” PLoS ONE 9, no. 6: e100724. 24967899 10.1371/journal.pone.0100724 PMC4072632

[brb370575-bib-0007] Akinkugbe, O. O . 1976. “Epidemiology of Hypertension and Stroke in Africa.” In Hypertension and Stroke Control in the Community, edited by S. Hatano , I. Shigematsu , and T. Strasser , 28–42. WHO.

[brb370575-bib-0008] Akinyemi, R. O. , B. Ovbiagele , O. A. Adeniji , et al. 2021. “Stroke in Africa: Profile, Progress, Prospects and Priorities.” Nature Reviews Neurology 17, no. 10: 634–656. 34526674 10.1038/s41582-021-00542-4 PMC8441961

[brb370575-bib-0009] Akpalu, A. , M. Gebregziabher , B. Ovbiagele , et al. 2019. “Differential Impact of Risk Factors on Stroke Occurrence Among Men Versus Women in West Africa.” Stroke 50, no. 4: 820–827. 30879432 10.1161/STROKEAHA.118.022786 PMC6433514

[brb370575-bib-0010] Alene, M. , M. A. Assemie , L. Yismaw , and D. B. Ketema . 2020. “Magnitude of Risk Factors and In‐Hospital Mortality of Stroke in Ethiopia: A Systematic Review and Meta‐Analysis.” BMC Neurology 20: 1–0. 32814556 10.1186/s12883-020-01870-6 PMC7437163

[brb370575-bib-0011] Al‐Jawaldeh, A. , and M. M Abbass . 2022. “Unhealthy Dietary Habits and Obesity: The Major Risk Factors Beyond Non‐Communicable Diseases in the Eastern Mediterranean Region.” Frontiers in Nutrition 9: 817808. 35369054 10.3389/fnut.2022.817808 PMC8970016

[brb370575-bib-0012] Avan, A. , H. Digaleh , M. Di Napoli , et al. 2019. “Socioeconomic Status and Stroke Incidence, Prevalence, Mortality, and Worldwide Burden: an Ecological Analysis from the Global Burden of Disease Study 2017.” BMC Medicine 17, no. 1: 191. 31647003 10.1186/s12916-019-1397-3 PMC6813111

[brb370575-bib-0013] Ayano, G. , K. Yohannis , M. Abraha , and B. Duko . 2019. “The Epidemiology of Alcohol Consumption in Ethiopia: A Systematic Review and Meta‐Analysis.” Substance Abuse Treatment, Prevention, and Policy 14, no. 1: 26. 31186050 10.1186/s13011-019-0214-5 PMC6558840

[brb370575-bib-0014] Aycock, D. M. , P. C. Clark , and S. Araya . 2019. “Measurement and Outcomes of the Perceived Risk of Stroke: A Review.” Western Journal of Nursing Research 41, no. 1: 134–154. 29243562 10.1177/0193945917747856 PMC5984120

[brb370575-bib-0015] Baye, M. , A. Hintze , C. Gordon‐Murer , et al. 2020. “Stroke Characteristics and Outcomes of Adult Patients in Northwest Ethiopia.” Frontiers in Neurology 11: 428. 32508740 10.3389/fneur.2020.00428 PMC7248259

[brb370575-bib-0016] Bereda, G. 2025. “Stroke Occurrence and Prevalence: Insights From a Comprehensive Review of Emerging Trends.” Preprint, Preprints.org, April 1. 10.20944/preprints202504.0028.v1.

[brb370575-bib-0017] Boden‐Albala, B. , J. Allen , E. T. Roberts , L. Bulkow , and B. Trimble . 2017. “Ascertainment of Alaska Native Stroke Incidence, 2005–2009: Lessons for Assessing the Global Burden of Stroke.” Journal of Stroke and Cerebrovascular Diseases 26, no. 9: 2019–2026. 28716585 10.1016/j.jstrokecerebrovasdis.2017.06.007

[brb370575-bib-0018] Boehme, A. K. , C. Esenwa , and M. S Elkind . 2017. “Stroke Risk Factors, Genetics, and Prevention.” Circulation Research 120, no. 3: 472–495. 28154098 10.1161/CIRCRESAHA.116.308398 PMC5321635

[brb370575-bib-0019] Boot, E. , M. S. Ekker , J. Putaala , et al. 2020. “Ischaemic Stroke in Young Adults: A Global Perspective.” Journal of Neurology, Neurosurgery & Psychiatry 91, no. 4: 411–417. 32015089 10.1136/jnnp-2019-322424

[brb370575-bib-0020] Brewin, J. N. , A. E. Smith , R. Cook , et al. 2020. “Genetic Analysis of Patients with Sickle Cell Anemia and Stroke Before 4 Years of Age Suggest an Important Role for Apoliprotein E.” Circulation: Genomic and Precision Medicine 13, no. 5: 531–540. 32924542 10.1161/CIRCGEN.120.003025

[brb370575-bib-0021] Bukhari, S. , S. Yaghi , and Z. Bashir . 2023. “Stroke in Young Adults.” Journal of Clinical Medicine 12, no. 15: 4999. 37568401 10.3390/jcm12154999 PMC10420127

[brb370575-bib-0022] Carnethon, M. R. , J. Pu , G. Howard , et al. 2017. “Cardiovascular Health in African Americans: A Scientific Statement from the American Heart Association.” Circulation 136, no. 21: e393–423. 29061565 10.1161/CIR.0000000000000534

[brb370575-bib-0023] Chen, W. , H. Ren , N. Wang , et al. 2021. “The Relationship Between Socioeconomic Position and Health Literacy Among Urban and Rural Adults in Regional China.” BMC Public Health 21: 1–0. 33731069 10.1186/s12889-021-10600-7 PMC7972343

[brb370575-bib-0024] de Havenon, A. , L. W. Zhou , K. C. Johnston , et al. 2023. “Twenty‐Year Disparity Trends in United States Stroke Death Rate by Age, Race/Ethnicity, Geography, and Socioeconomic Status.” Neurology 101, no. 5: e464–74. 37258298 10.1212/WNL.0000000000207446 PMC10401675

[brb370575-bib-0025] Demel, S. L. , S. Kittner , S. H. Ley , M. Mcdermott , and K. M. Rexrode . 2018. “Stroke Risk Factors Unique to Women.” Stroke 49, no. 3: 518–523. 29438077 10.1161/STROKEAHA.117.018415 PMC5909714

[brb370575-bib-0026] Elmberg Sjöholm, M. , G. Eriksson , A. Bii , J. Asungu , L. von Koch and S. Guidetti . 2021. “Living with Consequences of Stroke and Risk Factors for Unhealthy Diet‐Experiences Among Stroke Survivors and Caregivers in Nairobi, Kenya.” BMC Public Health 21: 1–2. 33726725 10.1186/s12889-021-10522-4 PMC7968355

[brb370575-bib-0027] Feigin, V. L. , M. Brainin , B. Norrving , et al. 2022. “World Stroke Organization (WSO): Global Stroke Fact Sheet 2022.” International Journal of Stroke 17, no. 1: 18–29. 34986727 10.1177/17474930211065917

[brb370575-bib-0028] Feigin, V. L. , M. O. Owolabi , V. L. Feigin , et al. 2023. “Pragmatic Solutions to Reduce the Global Burden of Stroke: a World Stroke Organization–Lancet Neurology Commission.” Lancet Neurology 22, no. 12: 1160–1206. 37827183 10.1016/S1474-4422(23)00277-6 PMC10715732

[brb370575-bib-0029] Fleischer, N. J. , and K. Sadek . 2024. “Arab, Middle Eastern, and North African Health Disparities Research: a Scoping Review.” Journal of Racial and Ethnic Health Disparities 11: 1–9. 38466512 10.1007/s40615-024-01972-8

[brb370575-bib-0030] Fowobaje, K. R. , A. P. Okekunle , J. Akinyemi , R. F. Afolabi , M. O. Owolabi , and O. M. Akpa . 2024. “Dominant Risk Factors for Stroke Among Africans: A Systematic Review and Meta‐Analysis.” African Journal of Biomedical Research 27, no. 4S: 7094–7118.

[brb370575-bib-0031] Gardener, H. , R. L. Sacco , T. Rundek , V. Battistella , Y. K. Cheung , and M. S. V. Elkind . 2020. “Race and Ethnic Disparities in Stroke Incidence in the Northern Manhattan Study.” Stroke; A Journal of Cerebral Circulation 51, no. 4: 1064–1069. 10.1161/STROKEAHA.119.028806 PMC7093213 32078475

[brb370575-bib-0032] Gardiner, F. W. , K. Rallah‐Baker , A. Dos Santos , et al. 2021. “Indigenous Australians Have a Greater Prevalence of Heart, Stroke, and Vascular Disease, Are Younger at Death, with Higher Hospitalisation and More Aeromedical Retrievals from Remote Regions.” EClinicalMedicine 42: 101181. 34765955 10.1016/j.eclinm.2021.101181 PMC8573152

[brb370575-bib-0033] Gidey, K. , and A. Hailu . 2023. “A Prospective Study of Stroke Characteristics, Risk Factors, and Mortality in a Tertiary Hospital of Northern Ethiopia.” International Journal of General Medicine 16: 5051–5061. 37942476 10.2147/IJGM.S433353 PMC10629449

[brb370575-bib-0034] Handebo, S. , S. Birara , A. Kassie , A. Nigusie , and W. Aleminew . 2020. “Smoking Intensity and Associated Factors among Male Smokers in Ethiopia: Further Analysis of 2016 Ethiopian Demographic and Health Survey.” BioMed Research International 2020, no. 1: 4141370. 32775418 10.1155/2020/4141370 PMC7396004

[brb370575-bib-0035] Huang, J. . 2024. “To What Extent, Which Factor Has the Most Significant Role Contributing to the Risk of Hypertension?.” Journal of Food Science, Nutrition and Health 2: 76–84.

[brb370575-bib-0036] Iadecola, C. , E. E. Smith , J. Anrather , et al. 2023. “The Neurovasculome: Key Roles in Brain Health and Cognitive Impairment: A Scientific Statement from the American Heart Association/American Stroke Association.” Stroke 54, no. 6: e251–71. 37009740 10.1161/STR.0000000000000431 PMC10228567

[brb370575-bib-0037] Jaberinezhad, M. , M. Farhoudi , S. A. Nejadghaderi , et al. 2022. “The Burden of Stroke and Its Attributable Risk Factors in the Middle East and North Africa Region, 1990–2019.” Scientific Reports 12, no. 1: 2700. 35177688 10.1038/s41598-022-06418-x PMC8854638

[brb370575-bib-0038] Johansson, T. , P. Fowler , W. E. Ek , A. Skalkidou , T. Karlsson , and Å. Johansson . 2022. “Oral Contraceptives, Hormone Replacement Therapy, and Stroke Risk.” Stroke 53, no. 10: 3107–3115. 35735009 10.1161/STROKEAHA.121.038659

[brb370575-bib-0039] Kandula, N. , M. Ahmed , S. Dodani , et al. 2019. “Cardiovascular Disease & Cancer Risk among South Asians: Impact of Sociocultural Influences on Lifestyle and Behavior.” Journal of Immigrant and Minority Health 21: 15–25. 28493115 10.1007/s10903-017-0578-4 PMC7646689

[brb370575-bib-0040] Kapral, M. K. , P. C. Austin , G. Jeyakumar , et al. 2019. “Rural‐Urban Differences in Stroke Risk Factors, Incidence, and Mortality in People with and Without Prior Stroke: The CANHEART Stroke Study.” Circulation: Cardiovascular Quality and Outcomes 12, no. 2: e004973. 30760007 10.1161/CIRCOUTCOMES.118.004973

[brb370575-bib-0041] Kim, J. , T. Thayabaranathan , G. A. Donnan , et al. 2020. “Global Stroke Statistics 2019.” International Journal of Stroke 15, no. 8: 819–838. 32146867 10.1177/1747493020909545

[brb370575-bib-0042] Kris‐Etherton, P. M. , K. S. Petersen , G. Velarde , et al. 2020. “Barriers, Opportunities, and Challenges in Addressing Disparities in Diet‐Related Cardiovascular Disease in the United States.” Journal of the American Heart Association 9, no. 7: e014433. 32200727 10.1161/JAHA.119.014433 PMC7428614

[brb370575-bib-0043] Lagranha, C. J. , T. L. A. Silva , S. C. A. Silva , et al. 2018. “Protective Effects of Estrogen against Cardiovascular Disease Mediated via Oxidative Stress in the Brain.” Life Sciences 192: 190–198. 29191645 10.1016/j.lfs.2017.11.043

[brb370575-bib-0044] Li, L. , C. A. Scott , and P. M Rothwell . 2022. “Association of Younger vs Older Ages With Changes in Incidence of Stroke and Other Vascular Events, 2002–2018.” Jama 328, no. 6: 563. 35943470 10.1001/jama.2022.12759 PMC9364129

[brb370575-bib-0045] Liu, Y. , H. Wang , B. Bai , et al. 2023. “Trends in Unhealthy Lifestyle Factors Among Adults With Stroke in the United States Between 1999 and 2018.” Journal of Clinical Medicine 12, no. 3: 1223. 36769871 10.3390/jcm12031223 PMC9917618

[brb370575-bib-0046] Marshall, I. J. , Y. Wang , S. Crichton , C. Mckevitt , A. G. Rudd , and C. D. A. Wolfe . 2015. “The Effects of Socioeconomic Status on Stroke Risk and Outcomes.” Lancet Neurology 14, no. 12: 1206–1218. 26581971 10.1016/S1474-4422(15)00200-8

[brb370575-bib-0047] Mehta, L. S. , G. P. Velarde , J. Lewey , et al. 2023. “Cardiovascular Disease Risk Factors in Women: The Impact of Race and Ethnicity: A Scientific Statement from the American Heart Association.” Circulation 147, no. 19: 1471–1487. 37035919 10.1161/CIR.0000000000001139 PMC11196122

[brb370575-bib-0048] Melak, A. D. , D. Wondimsigegn , and Z. D Kifle . 2021. “Knowledge, Prevention Practice and Associated Factors of Stroke Among Hypertensive and Diabetic Patients—A Systematic Review.” Risk Management and Healthcare Policy 14: 3295–3310. 34408515 10.2147/RMHP.S324960 PMC8364969

[brb370575-bib-0049] Miller, E. C. , and L. Leffert . 2020. “Stroke in Pregnancy: A Focused Update.” Anesthesia & Analgesia 130, no. 4: 1085–1096. 31124843 10.1213/ANE.0000000000004203 PMC7035913

[brb370575-bib-0050] Misgana, S. , M. A. Asemahagn , D. D. Atnafu , and T. F. Anagaw . 2023. “Incidence of Stroke and Its Predictors Among Hypertensive Patients in Felege Hiwot Comprehensive Specialized Hospital, Bahir Dar, Ethiopia, a Retrospective Follow‐Up Study.” European Journal of Medical Research 28, no. 1: 227. 37430339 10.1186/s40001-023-01192-6 PMC10332068

[brb370575-bib-0051] MJ Alqahtani, M. 2015. “Understanding the Sociocultural Health Belief Model Influencing Health Behaviors Among Saudi Stroke Survivors.” Neuroscience and Medicine 6, no. 04: 149–159.

[brb370575-bib-0052] Mohammed, A. S. , A. Degu , N. A. Woldekidan , F. Adem , and D. Edessa . 2021. “In‐Hospital Mortality and Its Predictors Among Stroke Patients in Sub‐Saharan Africa: A Systemic Review and Meta‐Analysis.” SAGE Open Medicine 9: 20503121211036789. 34377477 10.1177/20503121211036789 PMC8326621

[brb370575-bib-0053] Naftolin, F. , J. Friedenthal , R. Nachtigall , and L. Nachtigall . 2019. “Cardiovascular Health and the Menopausal Woman: The Role of Estrogen and When to Begin and End Hormone Treatment.” F1000Research 8: 1576. 10.12688/f1000research.15548.1 PMC6733383 31543950

[brb370575-bib-0054] Ng, R. , R. Sutradhar , Z. Yao , W. P. Wodchis , and L. C. Rosella . 2020. “Smoking, Drinking, Diet and Physical Activity—Modifiable Lifestyle Risk Factors and Their Associations With Age to First Chronic Disease.” International Journal of Epidemiology 49, no. 1: 113–130. 31329872 10.1093/ije/dyz078 PMC7124486

[brb370575-bib-0055] Nie, G. , X. Yang , Y. Wang , et al. 2022. “The Effects of Menopause Hormone Therapy on Lipid Profile in Postmenopausal Women: A Systematic Review and Meta‐Analysis.” Frontiers in Pharmacology 13: 850815. 35496275 10.3389/fphar.2022.850815 PMC9039020

[brb370575-bib-0056] Nott, M. , L. Wiseman , T. Seymour , S. Pike , T. Cuming , and G. Wall . 2021. “Stroke Self‐Management and the Role of Self‐Efficacy.” Disability and Rehabilitation 43, no. 10: 1410–1419. 31560230 10.1080/09638288.2019.1666431

[brb370575-bib-0057] Okonkwo, U. P. , E. M. Anyoke , E. Y. Ihegihu , et al. 2022. “Profile of Stroke Survivors Managed at the Physiotherapy Department of a Tertiary Teaching Hospital in Southeast Nigeria.” Tropical Journal of Medical Research 21, no. 1: 45–54.

[brb370575-bib-0058] Oyewole, O. O. , M. O. Ogunlana , C. A. Gbiri , K. S. Oritogun , and B. S. Osalusi . 2020. “Impact of Post‐Stroke Disability and Disability‐Perception on Health‐Related Quality of Life of Stroke Survivors: The Moderating Effect of Disability‐Severity.” Neurological Research 42, no. 10: 835–843. 32573376 10.1080/01616412.2020.1785744

[brb370575-bib-0059] Pandian, J. D. , Y. Kalkonde , I. A. Sebastian , C. Felix , G. Urimubenshi , and J. Bosch . 2020. “Stroke Systems of Care in Low‐Income and Middle‐Income Countries: Challenges and Opportunities.” Lancet 396, no. 10260: 1443–1451. 33129395 10.1016/S0140-6736(20)31374-X

[brb370575-bib-0060] Pantoja‐Ruiz, C. , R. Akinyemi , D. I. Lucumi‐Cuesta , et al. 2025. “Socioeconomic Status and Stroke: A Review of the Latest Evidence on Inequalities and Their Drivers.” Stroke 56, no. 3: 794–805. 39697175 10.1161/STROKEAHA.124.049474 PMC11850189

[brb370575-bib-0061] Park, J. H. , J. H. Moon , H. J. Kim , M. H. Kong , and Y. H. Oh . 2020. “Sedentary Lifestyle: Overview of Updated Evidence of Potential Health Risks.” Korean Journal of Family Medicine 41, no. 6: 365–373. 33242381 10.4082/kjfm.20.0165 PMC7700832

[brb370575-bib-0062] Peer, N. , L. Baatiema , and A. P Kengne . 2021. “Ischaemic Heart Disease, Stroke, and Their Cardiometabolic Risk Factors in Africa: Current Challenges and Outlook for the Future.” Expert Review of Cardiovascular Therapy 19, no. 2: 129–140. 33305637 10.1080/14779072.2021.1855975

[brb370575-bib-0063] Piano, M. R . 2017. “Alcohol's Effects on the Cardiovascular System.” Alcohol Research: Current Reviews 38, no. 2: 219. 28988575 10.35946/arcr.v38.2.06 PMC5513687

[brb370575-bib-0064] Powell, W. , J. Richmond , D. Mohottige , I. Yen , A. Joslyn , and G. Corbie‐Smith . 2019. “Medical Mistrust, Racism, and Delays in Preventive Health Screening Among African‐American Men.” Behavioral Medicine 45, no. 2: 102–117. 31343960 10.1080/08964289.2019.1585327 PMC8620213

[brb370575-bib-0065] Prabakaran, S. , A. Schwartz , and G. Lundberg . 2021. “Cardiovascular Risk in Menopausal Women and Our Evolving Understanding of Menopausal Hormone Therapy: Risks, Benefits, and Current Guidelines for Use.” Therapeutic Advances in Endocrinology and Metabolism 12: 20420188211013917. 34104397 10.1177/20420188211013917 PMC8111523

[brb370575-bib-0066] Prust, M. L. , R. Forman , and B. Ovbiagele . 2024. “Addressing Disparities in the Global Epidemiology of Stroke.” Nature Reviews Neurology 20, no. 4: 207–221. 38228908 10.1038/s41582-023-00921-z

[brb370575-bib-0067] Rizzoni, D. , M. Rizzoni , M. Nardin , et al. 2019. “Vascular Aging and Disease of the Small Vessels.” High Blood Pressure & Cardiovascular Prevention 26: 183–189. 31144248 10.1007/s40292-019-00320-w

[brb370575-bib-0068] Rountree, L. , Y. Fukuoka , K. Sagae , et al. 2024. “Perceived Susceptibility to and Severity of Cardiovascular Disease Is Associated With Intent to Change Behavior Among Women 25–55 Years Old.” Journal of Cardiovascular Nursing 10–97. 39454082 10.1097/JCN.0000000000001151

[brb370575-bib-0069] Sahle Adeba, T. , H. Mekonen , T. Alemu , T. Alate , and T. Melis . 2022. “Survival Status and Predictor of Mortality Among Adult Stroke Patients in Saint Paul's Hospital Millennium Medical College, Addis Ababa, Ethiopia.” SAGE Open Medicine 10: 20503121221112483. 35924142 10.1177/20503121221112483 PMC9340903

[brb370575-bib-0070] Saini, V. , L. Guada , and D. R Yavagal . 2021. “Global Epidemiology of Stroke and Access to Acute Ischemic Stroke Interventions.” Neurology 97, no. 20_Supplement_2: S6–16. 34785599 10.1212/WNL.0000000000012781

[brb370575-bib-0071] Salmantabar, P. , T. Abzhandadze , A. Viktorisson , M. Reinholdsson , and K. S. Sunnerhagen . 2022. “Pre‐Stroke Physical Inactivity and Stroke Severity in Male and Female Patients.” Frontiers in Neurology 13: 831773. 35359627 10.3389/fneur.2022.831773 PMC8963352

[brb370575-bib-0072] Seyoum Keflie, T. , A. Samuel , C. Lambert , D. Nohr , and H. K. Biesalski . 2018. “Dietary Patterns and Risk of Micronutrient Deficiencies: Their Implication for Nutritional Intervention in Ethiopia.” Journal of Nutrition Health and Food Science 6, no. 1: 1–16.

[brb370575-bib-0073] Shahbandi, A. , P. Shobeiri , S. Azadnajafabad , et al. 2022. “Burden of Stroke in North Africa and Middle East, 1990 to 2019: A Systematic Analysis for the Global Burden of Disease Study 2019.” BMC Neurology 22, no. 1: 279. 35896999 10.1186/s12883-022-02793-0 PMC9327376

[brb370575-bib-0074] Shaw, P. M. , V. Chandra , G. A. Escobar , N. Robbins , V. Rowe , and R. Macsata . 2018. “Controversies and Evidence for Cardiovascular Disease in the Diverse Hispanic Population.” Journal of Vascular Surgery 67, no. 3: 960–969. 28951154 10.1016/j.jvs.2017.06.111

[brb370575-bib-0075] Singh, V. , S. Prabhakaran , S. Chaturvedi , A. Singhal , and J. Pandian . 2017. “An Examination of Stroke Risk and Burden in South Asians.” Journal of Stroke and Cerebrovascular Diseases 26, no. 10: 2145–2153. 28579510 10.1016/j.jstrokecerebrovasdis.2017.04.036

[brb370575-bib-0076] Skolarus, L. E. , A. Sharrief , H. Gardener , C. Jenkins , and B. Boden‐Albala . 2020. “Considerations in Addressing Social Determinants of Health to Reduce Racial/Ethnic Disparities in Stroke Outcomes in the United States.” Stroke 51, no. 11: 3433–3439. 33104471 10.1161/STROKEAHA.120.030426 PMC7732185

[brb370575-bib-0077] Smythe, T. , G. Inglis‐Jassiem , T. Conradie , et al. 2022. “Access to Health Care for People With Stroke in South Africa: A Qualitative Study of Community Perspectives.” BMC Health Services Research 22, no. 1: 464. 35395847 10.1186/s12913-022-07903-9 PMC8993457

[brb370575-bib-0078] Sullivan, K. , and N. Thakur . 2020. “Structural and Social Determinants of Health in Asthma in Developed Economies: A Scoping Review of Literature Published Between 2014 and 2019.” Current Allergy and Asthma Reports 20: 1–2. 32030507 10.1007/s11882-020-0899-6 PMC7005090

[brb370575-bib-0079] Tang, R. 2025. “Effects of Problem‐Recognition Messages from Different Sources and Cues‐to‐Action on Promoting Corrective Efforts on Social Media.” Journal of Communication Management, ahead of print, April 21.

[brb370575-bib-0080] Thompson, S. C. , L. Nedkoff , J. Katzenellenbogen , M. A. Hussain , and F. Sanfilippo . 2019. “Challenges in Managing Acute Cardiovascular Diseases and Follow up Care in Rural Areas: A Narrative Review.” International Journal of Environmental Research and Public Health 16, no. 24: 5126. 31847490 10.3390/ijerph16245126 PMC6950682

[brb370575-bib-0081] Vellios, N. G. , and C. P Van Walbeek . 2018. “Self‐Reported Alcohol Use and Binge Drinking in South Africa: Evidence from the National Income Dynamics Study, 2014–2015.” South African Medical Journal 108, no. 1: 33. 10.7196/SAMJ.2017.v108i1.12615 29262976

[brb370575-bib-0082] Venketasubramanian, N. 2021. “Stroke Epidemiology in Oceania: A Review.” Neuroepidemiology 55, no. 1: 1–10. 33601397 10.1159/000512972

[brb370575-bib-0083] Venketasubramanian, N. , B. W. Yoon , J. Pandian , and J. C. Navarro . 2017. “Stroke Epidemiology in South, East, and South‐East Asia: A Review.” Journal of Stroke 19, no. 3: 286–294. 29037005 10.5853/jos.2017.00234 PMC5647629

[brb370575-bib-0084] Vincenzo, J. L. , S. K. Patton , L. L. Lefler , P. A. McElfish , J. Wei , and G. M. Curran . 2022. “A Qualitative Study of Older Adults' Facilitators, Barriers, and Cues to Action to Engage in Falls Prevention Using Health Belief Model Constructs.” Archives of Gerontology and Geriatrics 99: 104610. 34954649 10.1016/j.archger.2021.104610 PMC9344858

[brb370575-bib-0085] Wang, X. , C. Carcel , M. Woodward , and A. E. Schutte . 2022. “Blood Pressure and Stroke: A Review of Sex‐and Ethnic/Racial‐Specific Attributes to the Epidemiology, Pathophysiology, and Management of Raised Blood Pressure.” Stroke 53, no. 4: 1114–1133. 35344416 10.1161/STROKEAHA.121.035852

[brb370575-bib-0086] Xia, M. , K. Liu , J. Feng , Z. Zheng , and X. Xie . 2021. “Prevalence and Risk Factors of Type 2 Diabetes and Prediabetes Among 53,288 Middle‐Aged and Elderly Adults in China: A Cross‐Sectional Study.” Diabetes, Metabolic Syndrome and Obesity 14: 1975–1985. 10.2147/DMSO.S305919 PMC8104985 33976558

[brb370575-bib-0087] Yesilot, N. , J. Putaala , S. Z. Bahar , and T. Tatlisumak . 2017. “Ethnic and Geographical Differences in Ischaemic Stroke Among Young Adults.” Current Vascular Pharmacology 15, no. 5: 416–429. 28155625 10.2174/1570161115666170202161719

[brb370575-bib-0088] Zhang, X. , H. Lv , X. Chen , M. Li , X. Zhou , and X. Jia . 2023. “Analysis of Ischemic Stroke Burden in Asia from 1990 to 2019: Based on the Global Burden of Disease 2019 Data.” Frontiers in Neurology 14: 1309931. 38187147 10.3389/fneur.2023.1309931 PMC10770854

